# Engineering adoptive T cell therapy to co-opt Fas ligand-mediated death signaling in ovarian cancer enhances therapeutic efficacy

**DOI:** 10.1136/jitc-2021-003959

**Published:** 2022-03-09

**Authors:** Kristin G Anderson, Shannon K Oda, Breanna M Bates, Madison G Burnett, Magdalia Rodgers Suarez, Susan L Ruskin, Philip D Greenberg

**Affiliations:** 1 Clinical Research, Fred Hutchinson Cancer Research Center, Seattle, Washington, USA; 2 Immunology, University of Washington School of Medicine, Seattle, Washington, USA; 3 Ben Towne Center for Childhood Cancer Research, Seattle Children's Research Institute, Seattle, Washington, USA

**Keywords:** cell engineering, costimulatory and inhibitory T-Cell receptors, genital neoplasms, female, immunotherapy, adoptive, tumor microenvironment

## Abstract

**Background:**

In the USA, more than 50% of patients with ovarian cancer die within 5 years of diagnosis, highlighting the need for therapeutic innovations. Mesothelin (MSLN) is a candidate immunotherapy target; it is overexpressed by ovarian tumors and contributes to malignant/invasive phenotypes, making tumor antigen loss disadvantageous. We previously showed that MSLN-specific T cell receptor (TCR)-engineered T cells preferentially accumulate within established tumors, delay tumor growth, and significantly prolong survival in the ID8_VEGF_ mouse model that replicates many aspects of human disease. However, T cell persistence and antitumor activity were not sustained. We therefore focused on Fas/FasL signaling that can induce activation-induced cell death, an apoptotic mechanism that regulates T cell expansion. Upregulation of FasL by tumor cells and tumor vasculature has been detected in the tumor microenvironment (TME) of human and murine ovarian cancers, can induce apoptosis in infiltrating, Fas (CD95) receptor-expressing lymphocytes, and can protect ovarian cancers from tumor-infiltrating lymphocytes.

**Methods:**

To overcome potential FasL-mediated immune evasion and enhance T cell responses, we generated an immunomodulatory fusion protein (IFP) containing the Fas extracellular binding domain fused to a 4-1BB co-stimulatory domain, rather than the natural death domain. Murine T cells were engineered to express an MSLN-specific TCR (TCR_1045_), alone or with the IFP, transferred into ID8_VEGF_ tumor-bearing mice and evaluated for persistence, proliferation, cytokine production and efficacy. Human T cells were similarly engineered to express an MSLN-specific TCR (TCR_530_) alone or with a truncated Fas receptor or a Fas-4-1BB IFP and evaluated for cytokine production and tumor lysis.

**Results:**

Relative to murine T cells expressing only TCR_1045_, T cells expressing both TCR_1045_ and a Fas-4-1BB IFP preferentially persisted in the TME of tumor-bearing mice, with improved T cell proliferation and survival. Moreover, TCR_1045_/IFP^+^ T cells significantly prolonged survival in tumor-bearing mice, compared with TCR_1045_-only T cells. Human T cells expressing TCR_530_ and a Fas-4-1BB IFP exhibit enhanced functional activity and viability compared with cells with only TCR_530_.

**Conclusions:**

As many ovarian tumors overexpress FasL, an IFP that converts the Fas-mediated death signal into pro-survival and proliferative signals may be used to enhance engineered adoptive T cell therapy for patients.

## Introduction

More than 20,000 women are diagnosed with ovarian cancer annually, and >50% will die within 5 years.^1^ This mortality rate has changed little in the last 20 years, highlighting the need for therapy innovation.^2^ T cells engineered to selectively target proteins uniquely overexpressed in tumors represents a rapidly evolving strategy to control tumor growth without toxicity to healthy tissues. Mesothelin (MSLN) contributes to the malignant and invasive phenotype in ovarian cancer,^3 4^ and has limited expression in healthy cells,^5^ making it a candidate target^6^ Studies targeting MSLN with monoclonal antibodies,^7^ vaccination^8^ and chimeric antigen receptor (CAR)-expressing T cells^9^ have shown modest anti-tumor activity, validating MSLN as a viable target antigen.

T cells engineered to target MSLN (TCR_1045_) can preferentially accumulate within established tumors on adoptive transfer, delay tumor growth, and significantly prolong survival in the ID8_VEGF_ murine ovarian cancer model of established, disseminated disease.^10^ However, the previous study also revealed that elements in the tumor microenvironment (TME) limit engineered T cell persistence and the ability to eradicate cancer cells. The Fas death receptor is expressed on antigen-experienced T cells, and FasL expression in the tumor vasculature of human and murine ovarian cancers can induce apoptosis of Fas-expressing T cells,^11 12^ representing an obstacle to T cell accumulation in ovarian cancer.^12^ To overcome this T cell-evasion mechanism, we generated a panel of immunomodulatory fusion proteins (IFPs) containing the Fas extracellular binding domain fused to a 4-1BB co-stimulatory domain rather than the natural death domain.^13^ We previously demonstrated that this IFP can improve therapeutic efficacy of engineered T cells in mouse models of pancreatic cancer and acute myeloid leukemia,^13^ but the mechanism of in vivo action and the potential broader utility of this strategy remain undefined.

We have now confirmed our hypothesis that T cells expressing TCR_1045_ and a Fas-4-1BB IFP would persist better within ID8_VEGF_ ovarian tumors, with less apoptosis and potentially enhanced T cell proliferation in situ. IFP^+^ T cells persisted longer than cells only expressing TCR_1045_ when FasL was present on tumor cells. T cells engineered with TCR_1045_ and the Fas-4-1BB IFP were more therapeutically effective, improving survival of tumor-bearing mice without on-target, off-tumor toxicity. We have also validated that human CD8 T cells expressing a MSLN-specific TCR and a Fas-4-1BB IFP exhibit enhanced functional activity compared with T cells expressing only the TCR or the TCR and a truncated Fas receptor. These studies validate that a rationally designed fusion protein can address Fas-mediated apoptotic T cell death, otherwise an obstacle to the efficacy of engineered T cell therapy.

## Methods

### Cell lines

ID8_VEGF_ cells, which were transduced to overexpress vascular endothelial growth factor (VEGF) to recapitulate the elevated levels observed in human disease,^14^ were a gift from Dr Matthias Stephan in 2014. ID8 cells were cultured in DMEM (Gibco) containing 10% Fetal Bovine Serum (FBS, Hyclone), 1% Penn/Strep (Gibco), and 0.1% Insulin-Transferrin-Selenium (Sigma). ID8_VEGF_ cell lines were passaged fewer than 10 times before use in experiments. PLAT-E cells (ATCC) passaged fewer than 35 times were used for retrovirus production for all T cell transductions. All cell lines were confirmed negative for mycoplasma prior to use. Cell lines were not authenticated in the past year.

### Mouse strains

All mice were co-housed and provided with nesting material for enrichment. Cages were maintained on the same row of the multi-tier caging system. 5×10^6^ ID8_VEGF15_ cells were injected i.p. into >8-week-old female C57BL/6J mice (Jackson Laboratories).

### ID8_VEGF_ FasL CRISPR KO

ID8_VEGF_ cells were edited using CRISPR knockout technology by Synthego. Briefly, founder ID8_VEGF_ cells (mycoplasma-free) were sequenced and synthetic single guide RNA (sgRNA) was designed to target FasL (guide target sequence: CTCCTTTGGTCCGGCCCTCT). Specific guide RNA was complexed with *Streptococcus pyogenes* Cas9 (PAM motif: AGG) into a ribonucleoprotein and delivered into ID8_VEGF_ cells by electroporation. PCR-amplification and Sanger sequencing was used to sequence the transfected cells to verify knockout efficiency. Cells from the edited pool were seeded using single cell dilution and expanded to establish clonal lines. Clones were sequenced to verify the clone contained a homozygous edit and that the progeny were derived from a single cell, and a single clone (E9) was selected for experiments.

### Cytotoxicity assays

Naïve P14 T cells were activated for 72 hours with anti-CD3 and CD28 antibodies, then cultured alone or co-cultured with tumor cells for 18–24 hours in 24-well tissue culture plates. T cells were then gently removed and transferred to FACS tubes for intracellular cleaved-caspase 3 (CC3) staining. The BD Fix/Perm kit (cat: 554714) was used for staining with anti-CC3.

### Retroviral constructs

The codon-optimized murine Vβ9 and Vα4 TCR chains, connected by a porcine teschovirus-1 2A element (“P2A”; Life technologies), recognize the Msln_406-414_ epitope presented in H2-D^b^, and were cloned from the Mig-R1 retroviral vector^16^ into the pENTR vector and subsequently Gateway cloned into the pMP71 retroviral vector for all TCR_1045_ studies. A construct encoding Fas-4-1BB_tm_ and TCR_1045_ was ordered from GeneArt (ThermoFisher) in the pDONR221 vector and gateway cloned into the pMP71 retroviral vector for all TCR_1045_ studies.

### TCR transduction of T cells

Murine T cell transduction has been described previously^10^ and can be accessed at DOI: dx.doi.org/10.17504/protocols.io.smrec56. Briefly, PLAT-E cells were transfected with DNA encoding TCR_1045_ or TCR_1045_/Fas-4-1BB_tm_, the media was replaced at 24 hours, and the viral supernatant used for T cell transduction 48 and 72 hours. Splenic T cells isolated from P14 Thy1.1^+^ mice were stimulated with anti-CD3 and anti-CD28 antibodies in the presence of IL-2 and transduced with retroviral supernatant by spinfection in polybrene at 24 and 48 hours after activation. T cell restimulation has been described previously^10^ and can be access at DOI: dx.doi.org/10.17504/protocols.io.spqedmw. Briefly, Thy1.2^+^ splenocytes were irradiated and pulsed with Msln_406-414_ peptide for >90 min to prepare APCs. Transduced T cells were co-cultured with peptide-pulsed APCs for 5–7 days in the presence of IL-2. T cells were screened by flow cytometry for transduction efficiency 5 days after activation or restimulation.

Human T cell transduction and rapid expansion protocol have been descried previously and can be accessed at DOI: dx.doi.org/10.17504/protocols.io.sxvefn6. Briefly, 293 T cells were transfected with DNA encoding TCR_HIVgag_, TCR_530_, TCR_530_/FasTr, TCR_530_/Fas_tm_-4-1BB, or TCR_530_/Fas-4-1BB_tm_, the media was replaced at 24 hours, and the viral supernatant used for T cell transduction at 48 hours. CD8^+^ T cells were thawed and activated with anti-CD3 and -CD28 beads in the presence of IL-2 and transduced by lentiviral supernatant by spinfection in polybrene between 8 and 24 hours after activation. Cells were expanded by culturing with irradiated PBMC and LCL cells in the presence of anti-CD3 antibody (OKT3) and IL-2. T cells were screened by flow cytometry for transduction efficiency and used for experiments 7 days after activation or restimulation.

### T cell isolation

Tumors were dissociated in 3 mL of RPMI with 10% FBS using the m_imptumor_01 setting on a gentleMACS dissociator (Miltenyi Biotec). Samples were transferred to a conical tube with 35 mL of Collagenase (RPMI containing 1% HG solution, 0.1% MgCl2, 0.1%CaCl2, 5% FBS, and 20,000U of Collagenase Type IV) and mixed on a MACSmix rotator at 37°C for 10 min. Samples were filtered through cell strainers (70 µm Falcon) and lymphocytes were purified on a 44/67% percoll gradient (800x*g* at 20°C for 20 min). Spleens were dissociated by mechanical separation through a cell strainer and ACK lysis (Gibco, cat: A10492-01) was performed to remove red blood cells.

### Flow cytometry

MSLN_406-414_/H2-D^b^ and MSLN_530-538_/HLA-A*02.01 tetramers conjugated to APC were prepared by the Fred Hutch Immune Monitoring Core. All cells were stained with LIVE/DEAD fixable Aqua (405 nm, cat: L34966) or fixable Blue (350 nm, cat: L23105) in 1 x DPBS prior to surface or intracellular staining. UltraComp eBeads (eBioscience, cat: 01–2222) were used for all compensation. For ex vivo experiments, cells from either untreated mice or endogenous CD44^-^ CD62L^+^ CD8^+^ T cells from the spleen of treated mice were used for negative controls and gating. For in vitro experiments, fluorescence minus one or irrelevant engineered T cells were used for negative controls and gating. For murine FasL staining, cells were blocked with human and mouse serum and then stained with purified Armenian hamster anti-mouse CD178 (clone: MFL3) followed by secondary staining with polyclonal goat anti-Armenian hamster IgG PE (Invitrogen). The eBioscience Fix/Perm kit (cat: 00-5523-00) was used for staining with anti-Ki67 (cat: 51–36524X) with a mouse IgG_1_ isotype as a control (cat: 51–35404X). Cells were resuspended in FACS buffer (1 x DPBS containing 2% FBS Hyclone and 0.72% 0.5M EDTA) or 0.5% Paraformaldehyde and acquired with an LSR2-2, Fortessa or Symphony Instrument (BD).

### Intracellular cytokine stimulation

The stimulation protocol for intracellular cytokine staining has been described previously^10^ and can be found at DOI: dx.doi.org/10.17504/protocols.io.sqdeds6. Briefly, cells were treated with protein transport inhibitor containing Brefeldin A (GolgiPlug) and plated at 1×10^6^ cells per well in a flat-bottom 96-well plate. Cells were stimulated for 5 hours at 37°C with T cell media or 1 mg/mL of Msln_406-414_ peptide (GQKMNAQAI; murine T cells) or Msln_530-538_ (VLPLTVAEV; human T cells). Peptides were ordered from ELIM peptide (>80% purity). The BD Fix/Perm kid was used for intracellular staining. In some experiments, cells were fixed in 0.5% paraformaldehyde until data acquisition.

### Cell proliferation

The Click-iT Plus EdU imaging kit (cat: C10640) was reconstituted at 10 mM in DMSO and used to analyze in situ cell proliferation. For in vitro experiments, 3×10^6^ cells were plated with 5 µL of the 10 mM EdU solution and incubated for 24 hours. Cells were treated with the eBioscience Fix/Perm kit and subsequently stained using Click-iT Plus EdU imaging kit reagents: Click-iT reaction buffer, copper protectant, Alexa Fluor picolyl azide (AF647 fluorescent), and Click-iT reaction buffer additive. Samples were analyzed by flow cytometry within 4 hours. For in vivo EdU experiments, EdU was diluted to 5 mg/mL of saline. Mice were injected i.p. with 50 mg EdU/kg and cells were isolated 24 hours later by enzymatic digestion. Cells were stained using the Click-iT Plus EdU imaging kit and analyzed by flow cytometry within 4 hours.

### Quantitative PCR

3×10^6^ transduced T cells were resuspended in RLT lysis buffer (supplemented with b-ME) and RNA extracted with the Qiagen RNeasy Plus RNA isolation kit. RNA integrity was analyzed on Tape Station analyzer and cDNA was generated using iScript Reverse Transcription Supermix for RT-qPCR (Biorad). qPCR was run using qPCR SYBR Green Assay with mouse BCL2 PrimePCR assay (Biorad) and with RPL13a reference gene (Primers: Fwd.: TTCTCCTCCAGAGTGGCTGT, Rev.: GGCTGAAGCCTACCAGAAAG) on the 384 well ABI QuantStudio5 instrument. The delta delta CT method was used for analysis.

### Adoptive immunotherapy

ID8_VEGF_-tumor-bearing mice received either engineered T cells (1×10^7^, transduced and re-stimulated in vitro) or engineered T cells (1×10^7^) transduced without restimulation in vitro but then stimulated in vivo with 5×10^7^ peptide-pulsed irradiated splenocytes as a vaccine. Cell infusions were followed by IL-2 (2×10^4^ IU, s.c.) daily for 10 days to promote T-cell expansion and survival. For therapy with serial T-cell infusions, mice received this same treatment protocol every 2 weeks. In indicated experiments, treated mice received one dose of Cyclophosphamide (180 mg/kg) i.p. to lymphodeplete hosts approximately 6–8 hours prior to only the first T-cell transfer. Mice were randomized by one investigator and treated by a second. Power analysis guided enrolment numbers to power the study for a large effect (>50% increase in median overall survival).

### Impedance cytotoxicity assay

1×10^4^ OVCAR3 tumor cells were plated on gold-electrode E-plates (xCELLiegence, Agilent), plates were connected to the xCELLigence RTCA MP instrument and allowed to adhere for 24 hours. When cells reached logarithmic growth, T cells were added at varying E:T ratios (5:1 to 0.625:1) in triplicate. Irrelevant T cells (TCR_HIVgag_) were used to calculate tumor-specific killing above background.

### Immunohistochemistry

Histology preparation has been described previously^10^ and can be found at DOI: dx.doi.org/10.17504/protocols.io.sppedmn. Briefly, harvested tissues were fixed in 10% neutral buffered formalin for at least 72 hours, embedded in paraffin, sectioned (4 um) and stained with hematoxylin and eosin or primary antibodies for markers of interest using a Leica Bond Instrument. Following antigen retrieval, slides were blocked with Leica Bond Peroxide Block and then 10% normal goat serum, stained with primary antibodies for 30 or 60 min, and Leica Bond polymer was applied. Leica Bond Mixed Refine (DAB) detection was performed and a Leica hematoxylin counterstain was added. Slides were cleared with xylene, mounted, and scanned in brightfield (20 x) using the Aperio ScanScope AT slide scanner. Digital images were imported into Aperio eSlide manager.

### Halo analysis

Images were analyzed using HALO (Indica Labs). Cells were determined based on thresholds set for nuclear size, segmentation, contrast threshold, and maximum cytoplasm radius. FasL and CD31 expression was analyzed using positive staining set by the minimum optical density (OD) above the background (set on regions of non-specific staining). Co-localization was analyzed by using image registration to align both FasL and CD31 stains and then using nearest neighbor analysis. Cell location within tumors was evaluated by concentric partitioning analysis with Cytonuclear v2.0.9 per partition. Lung tissue was annotated, and the total number of cells was identified using cell by cell analysis. CD8^+^ or CD3^+^ cells were identified by positive staining set by the minimum optical density (OD) above the background. MSLN expression analysis was performed by annotating the tumor region and using the Area Quant analysis to determine the percentage of area with positive MSLN staining set by the minimum OD above the background.

### Statistics

The Student’s t-test was used to compare normally distributed two-group data. A one-way Anova with post hoc analysis pairwise for multiple comparisons was used to compare data from experiments with more than two groups. Survival curve analysis was performed using the Log-rank (Mantel-Cox) and Gehan-Breslow-Wilcoxon tests. All error bars represent SD.

## Results

### FasL expression on ovarian tumor cells and effector T cells induces CD8 T cell death

**Figure 1:**
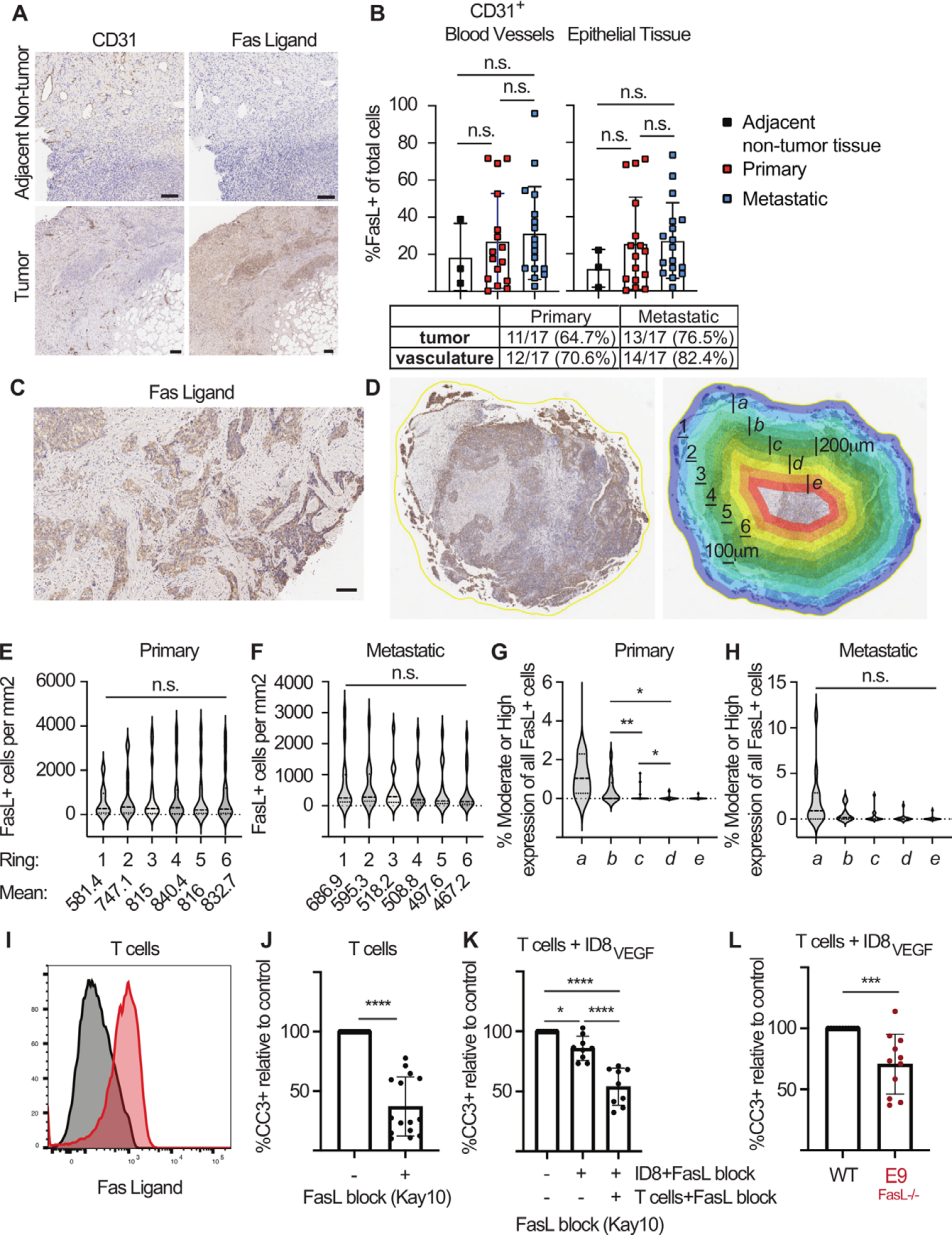
Fas ligand signaling from T cells and ID8_VEGF_ tumor cells induce death in effector CD8 T cells. (A) Immunohistochemistry (IHC) staining for CD31 and Fas Ligand in human high-grade serous ovarian cancer. Images are representative of 17 primary and 17 metastatic patient samples. Scale bar=500 µm. (B) Halo quantification of FasL^+^ cells in CD31^+^ vasculature or tumor epithelium. Samples were considered positive if >10% of cells were FasL^+^. Two-way ANOVA for multiple comparisons. n.s.=not significant. (C) Representative IHC staining for FasL, with higher intensity staining near the tumor edge. Scale bar=100 µm. (D) Example of concentric partitioning analysis in Halo. Consecutive 100 (denoted ‘1–6’) or 200 µm (denoted ‘a-e’) rings are annotated on each sample and are referred to in (E–H). (E, F) Quantification of FasL^+^ cells in 100 µm concentric rings from the outer edge of the sample to the center. Concentric rings, as in (D), were annotated as Ring 1 (0–100 µm), ring 2 (100–200 µm), ring 3 (200–300 µm), ring 4 (300–400 µm), ring 5 (400–500 µm), ring 6 (500–600 µm) on primary (E, F) metastatic tumor samples. Mixed-effects analysis for multiple comparisons. (G, H) Percent of FasL^+^ cells with moderate or high staining intensity in 200 µm concentric rings from the outer edge of the sample to the center. Concentric rings, as in (D), were annotated as Ring 1 (0–200 µm), ring 2 (200–400 µm), ring 3 (400–600 µm), ring 4 (600–800 µm), ring 5 (800–1000 µm) on of G) primary and (H) metastatic tumor samples. Mixed-effects analysis for multiple comparisons. *P<0.5, **p<0.01. (I) FasL flow cytometry staining on activated CD8 T cells 7 days after activation with peptide-pulsed irradiated splenocytes. Results are representative of 4 independent experiments. (J) Cleaved Caspase 3 (CC3) flow cytometry staining in activated CD8 T cells 3 days after culture with or without pre-treatment with anti-FasL blocking antibody (clone: Kay10). Data are displayed as a percentage of CC3^+^ staining relative to untreated T cells. Cumulative data from five independent experiments. Student’s unpaired t-test. ****P<0.0001. (K) CC3 flow cytometry staining in activated CD8 T cells 3 days after culture alone or coculture with ID8_VEGF_ tumor cells. T cells or tumor cells were pre-treated with FasL (Kay10) blocking antibody as indicated. Data displayed as a percentage relative to T cells cocultured with ID8_VEGF_ tumor cells without FasL blockade. Cumulative data from three independent experiments. One-way ANOVA for multiple comparisons. *P=0.0353; ****p<0.0001 (L) CC3 flow cytometry staining in activated CD8 T cells 3 days after co-culture with wild type ID8_VEGF_ or CRISPR edited ID8_VEGF_^FasL-/-^ tumor cells (E9 clone). Data displayed as a percentage relative to T cells co-cultured with wild type ID8_VEGF_ tumor cells. Cumulative data from four independent experiments. Student’s unpaired t-test. ***p=0.0008. All error bars indicate SD. ANOVA, analysis of variance; ns, not significant.

FasL expression by solid tumors promotes tumor metastasis^17^ and immune evasion.^18^ FasL is overexpressed in ovarian tumor vasculature^12^ and has higher expression in the ovarian TME than in normal ovarian tissue.^19^ We evaluated the distribution of FasL expression in high-grade serous ovarian cancer. Serial sections from matched primary and metastatic high-grade serous tumor samples were stained for the vascular marker CD31 and FasL (figure 1A–B); merged images were analyzed by nearest-neighbor Halo infiltration analysis. Above-background FasL expression was detected in the vasculature of 12/17 primary and 14/17 metastatic tumors, consistent with previous reports,^12 19^ but was also detected in the epithelial tumor regions of 11/17 primary and 13/17 metastatic tumors. Imaging revealed heterogenous FasL expression in patient samples (figure 1C), and we used concentric partitioning with cytonuclear Halo image analysis (figure 1D) to further evaluate the location of FasL^+^ cells and the intensity of FasL expression in tumors. Analysis of 100 µm concentric regions (denoted in figure 1D–F as numbered ‘rings’) revealed similar numbers of FasL^+^ cells in epithelial regions of primary (figure 1E) and metastatic tumors (figure 1F). While we observed a trend toward fewer FasL^+^ cells in the more central regions of metastatic tumors, this did not reach statistical significance. Analysis of FasL expression in 200 µm concentric regions (denoted in figure 1D and G–H as ‘rings a-e*’*) revealed the highest levels often found near the tumor edge (figure 1G–H). Thus, T cells infiltrating ovarian tumors can encounter FasL death signals not only while exiting the vasculature but also on encountering and then infiltrating epithelial tumors.

FasL is also expressed on activated T cells (figure 1I).^20 21^ To evaluate if FasL signaling from fellow T cells can induce fratricide, we activated wild-type P14 CD8 T cells, expressing a TCR (TCR_gp33_) specific for the gp33-41 epitope of lymphocytic choriomeningitis virus (LCMV), with anti-CD3 and CD28 antibodies in vitro for 8 hours. We then treated the T cells with media or a FasL blocking antibody (Kay-10 clone) and stained for cleaved caspase 3 (CC3) 3 days later. FasL blockade significantly reduced CC3 expression (figure 1J), suggesting that activated T cells engage in Fas/FasL apoptosis-inducing signaling. To determine if FasL expression on ID8_VEGF_ tumor cells also induces CD8 T cell death, we co-cultured wild type ID8_VEGF_ cells with P14 CD8 T cells previously activated in vitro with anti-CD3 and CD28 antibodies (±pretreatment with FasL-blocking antibody), and stained for CC3 in T cells 3 days later. Significantly decreased CC3 staining was observed when tumor-cell FasL signaling was inhibited by the antibody (figure 1K); concurrent T cell FasL blockade further decreased CC3 levels. To verify these results suggesting tumor cell-induced apoptosis of T cells, we used CRISPR/Cas9 targeting to generate a FasL knockout ID8_VEGF_ cell line (ID8_VEGF_^FasL-/-^; denoted E9; online supplemental figure 1). CC3 staining was reduced in activated T cells co-cultured with E9 ID8_VEGF_^FasL-/-^ cells, but not in activated wild-type ID8_VEGF_ cells (figure 1L). Thus, effector CD8 T cells are susceptible to FasL signals from both ID8_VEGF_ tumor cells and fellow CD8 T cells.

10.1136/jitc-2021-003959.supp1 Supplementary data

### TCR_1045_/Fas-4-1BB IFP^+^ T cells exhibit enhanced survival *in vivo*

**Figure 2:**
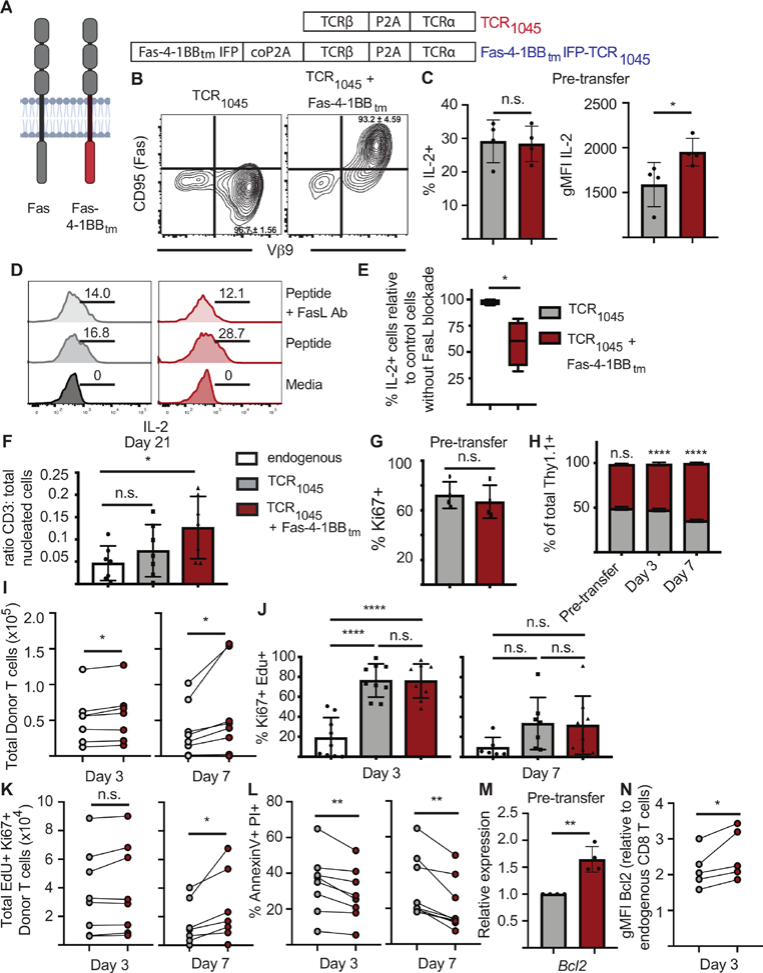
Expression of a Fas-IFP enhances accumulation of CD8 T cells in vivo (A) Schematic of monomeric endogenous Fas receptor, a Fas-4-1BB_tm_ immunomodulatory fusion protein, and vectors encoding TCR_1045_ or TCR_1045_ and a Fas-4-1BB_tm_ immunomodulatory fusion protein (IFP). Genes are separated by P2A or codon-optimized (co) P2A elements to ensure equimolar expression. (B) CD95 (Fas) and Vβ9 expression on transduced P14 Thy1.1^+^ CD8 T cells. T cells were transduced with retroviral vectors containing TCR_1045_ or TCR_1045_ and Fas-4-1BB_tm_ IFP and re-stimulated with irradiated splenocytes pulsed with Msln_406-414_ in the presence of IL-2. Cells were screened by flow cytometry 7 days after restimulation. Data representative of >5 independent experiments. Quadrant values indicate mean and SD from five independent experiments. (C) Intracellular cytokine staining for IL-2 production by P14 Thy1.1^+^ CD8 T cells expressing either TCR_1045_ (gray) or TCR_1045_ and Fas-4-1BB_tm_ IFP (red) after 5-hour stimulation with Msln_406-414_ peptide presented as a percent of engineered T cells (left panel) and as geometric mean fluorescence intensity (gMFI, right panel). Cumulative data from three independent experiments (1–3 technical replicates per experiment). Unpaired t-test, **p=0.0040. n.s.=not significant. (D, E) In vitro intracellular cytokine staining for IL-2 production by P14 Thy1.1^+^ TCR_1045_ (gray) or TCR_1045_/Fas-4-1BB_tm_ IFP (red) CD8 T cells, with or without FasL blockade (clone: Kay10), after 5 hour stimulation with Msln_406-414_ peptide. (D) Representative flow cytometry plots from one experiment. The average percentage of IL-2^+^ cells is displayed for each condition (samples run in triplicate). (E) Data are displayed as a percent difference between cells treated with FasL blocking Ab (clone: Kay10) compared with cells treated with isotype control Ab. Cumulative data from four independent experiments. Paired t-test. *P<0.05. (F) Immunohistochemistry (IHC) staining for CD3^+^ T cells in ID8_VEGF_ tumors 21 days after T cell transfer. P14 Thy1.1^+^ CD8 T cells expressing TCR_1045_ or TCR_1045_ and Fas-4-1BB_tm_ IFP were injected i.p. into ID8_VEGF_ tumor-bearing mice. Mice were pre-treated with 180 mg/kg cyclophosphamide >6 hours prior to cell transfer. All mice received 1×10^7^ engineered T cells, 5×10^7^ irradiated splenocytes pulsed with Msln_406-414_ peptide, and daily IL-2 s.c. for 10 days (1×10^4^ IU). CD3 staining in untreated mice (white bar) included as a reference for endogenous T cell infiltration. Cumulative data from three independent experiments, n=7 per group. One-way ANOVA Tukey’s multiple comparisons test, *p=0.0442. n.s.=not significant G) Intracellular flow cytometry staining for Ki67 expression in Thy1.1^+^ CD8^+^ Vβ9^+^ T cells 7 days after in vitro restimulation of engineered T cells. Data from four independent experiments. Unpaired t-test. H–N) Thy1.1^+^ or Thy1.1/1.2^+^ P14 T cells expressing TCR_1045_ (gray) or TCR_1045_ and Fas-4-1BB_tm_ IFP (red) were co-transferred at a 1:1 ratio into ID8_VEGF_ tumor-bearing mice with 5×10^7^ irradiated splenocytes pulsed with Msln_406-414_ peptide, and daily s.c. IL-2 (1×10^4^ IU). at 3 or 7 days after co-transfer, T cells were isolated from tumors, enumerated and evaluated by flow cytometry for indicated markers. (H) Proportion of donor T cells within ID8_VEGF_ tumors after co-transfer. Data pooled from three independent experiments, n=8 (day 3) or n=7 (day 7) mice per group. Two-way ANOVA for multiple comparisons. n.s. (TCR_1045_ from pre-transfer to D3 and TCR_1045_/Fas-4-1BB_tm_ IFP pre-transfer to D3). ****p<0.0001 (TCR_1045_ from D3 to D7 and TCR_1045_/Fas-4-1BB_tm_ IFP from D3 to D7). (I) Quantification of intratumoral donor T cells. Counts of the cotransferred T cell populations from each individual mouse are connected with a line. Paired t-test. *P<0.05. (J) Flow cytometry staining of intratumoral endogenous and donor T cells three or 7 days after transfer for Ki67 expression and EdU incorporation. EdU was injected i.p. into mice 24 hours prior to T cell isolation. 1-way ANOVA for multiple comparisons. ****p<0.0001. n.s.=not significant. (K) Quantification of EdU^+^ Ki67^+^ intratumoral donor T cells. Counts of the cotransferred cell populations from each individual mouse are connected with a line. Paired t-test. *P<0.05. (L) Flow cytometry staining of intratumoral donor T cells three or 7 days after transfer for Annexin V and Propidium Iodide (PI). Cell populations from the same mouse are connected with a line. Data from three independent experiments, n=8 (day 3) or n=9 (day 7). Paired t-test. **P<0.01. (M) qPCR for *Bcl2* expression in TCR_1045_ (gray) or TCR_1045_/Fas-4-1BB_tm_ IFP (red) T cells 8 days after restimulation in vitro. Data pooled from four independent experiments. Unpaired t-test **p=0.0016. (M) Flow cytometry staining of intratumoral engineered T cells 3 days after transfer for intracellular Bcl2. Data are displayed relative to expression in endogenous CD8 T cells from the spleen of the same mouse. Cell populations from the same mouse are connected with a line. Data from three independent experiments, n=5. Paired t-test. *P=0.0376. All error bars represent SD. ANOVA, analysis of variance.

We previously isolated the high-affinity murine TCR_1045_ specific for the MSLN_406-414_ epitope and demonstrated that CD8 T cells transduced with TCR_1045_ mediate antitumor activity against ovarian and pancreatic cancers without toxicity to normal tissues.^10 16^ We also previously developed an IFP containing the Fas extracellular binding domain and a 4-1BB co-stimulatory domain; expression of this fusion protein improved the efficacy of T cell therapy in mouse models of acute myeloid leukemia and pancreatic cancer.^13^ We now tested whether the Fas-4-1BB_tm_ IFP could improve therapeutic activity in a mouse model of ovarian cancer in which FasL/Fas signaling is known to reduce T-cell efficacy.^12^ Naive transgenic P14 CD8 T cells were activated with anti-CD3 and CD28 antibodies and transduced with either a bi-cistronic vector encoding the alpha and beta chains of TCR_1045_, or a tri-cistronic vector encoding both TCR_1045_ chains and a Fas-4-1BB_tm_ IFP (TCR_1045_/Fas-4-1BB_tm_, figure 2A), linked by P2A elements to ensure equimolar expression. P14 T cells engineered with either TCR_1045_ or TCR_1045_/Fas-4-1BB_tm_ expressed similar levels of the Vβ9 component of TCR_1045_, but only TCR_1045_/Fas-4-1BB_tm_ T cells expressed high levels of Fas, reflecting high levels of transduced IFP (figure 2B). We previously reported that Fas-4-1BB_tm_-expressing T cells produce increased levels of IL-2,^13^ and we validated that the TCR_1045_/Fas-4-1BB_tm_ fusion protein also increased IL-2 production by transduced T cells (figure 2C). Blocking FasL during in vitro stimulation reduced IL-2 production by TCR_1045_/Fas-4-1BB_tm_ T cells, but not TCR_1045_ cells, suggesting FasL signals promote IL-2 production by IFP^+^ T cells (figure 2D–E).

We also previously showed that TCR_1045_-T cells infiltrate ID8_VEGF_ tumors, but that antitumor activity is limited by low persistence.^10^ To evaluate if TCR_1045_/Fas-4-1BB_tm_ T cells persist better in mouse ovarian tumors than control TCR_1045_ cells, T cells were transferred into ID8_VEGF_ tumor-bearing mice, following an established protocol.^10^ Briefly, 5×10^6^ ID8_VEGF_ cells were injected intraperitoneally (i.p.) into 8 week-old female C57Bl/6 mice. After tumor nodules were readily detectable by high resolution ultrasound (>6 weeks after tumor injection), mice were treated with cyclophosphamide ≥6 hours to render the host lymphopenic, and then received 1×10^7^ engineered T cells and a vaccine of 5×10^7^ irradiated splenocytes pulsed with MSLN_404-416_ peptide to enhance engraftment.^10^ Prior to transfer,>93% of the transduced T cells expressed TCR_1045_ or TCR_1045_/Fas-4-1BB_tm_, as indicated by staining for the Vβ9 chain of TCR_1045_ (figure 2B). By twenty-one days after T cell transfer, the number of T cells in TCR_1045_ T cell-engrafted tumors was not significantly greater than the number of endogenous T cells in tumors from untreated mice, but 3-fold more T cells persisted in TCR_1045_/Fas-4-1BB_tm_ T cell-treated vs untreated tumors (figure 2F). As co-stimulation through 4-1BB promotes T cell proliferation and survival,^22 23^ enhanced persistence of intratumoral TCR_1045_/Fas-4-1BB_tm_ T cells could be due to increased T cell proliferation, reduced T cell death or both. To evaluate T cell proliferation, we assessed Ki67 expression and EdU incorporation by tumor-infiltrating engineered T cells. Congenically distinct (Thy1.1^+^ or Thy1.1^+^Thy1.2^+^) P14 T cells were transduced with TCR_1045_ or TCR_1045_/Fas-4-1BB_tm_ and equal numbers were co-transferred into tumor-bearing mice. Prior to T cell transfer, a similar proportion of TCR_1045_ and TCR_1045_/Fas-4-1BB_tm_ T cells expressed Ki67 (figure 2G), suggesting expressing Fas-4-1BB_tm_ does not change the proportion of cycling, activated cells in vitro. At 3 or 7 days post-transfer, TCR_1045_/Fas-4-1BB_tm_ T cells preferentially accumulated within tumors (figure 2H–I). Although there was no difference in the proportion of Ki67^+^Edu^+^ cells at either time point (figure 2J), 1.7-fold more Ki67^+^EdU^+^ TCR_1045_/Fas-4-1BB_tm_ T cells were present 7 days post-transfer (figure 2K). To address enhanced proliferation in situ and/or reduced cell death in the TCR_1045_/Fas-4-1BB_tm_ T cell population, we stained intratumoral T cells with Annexin V and Propidium Iodide (PI). Three- and 7 days post-transfer, 1.2-fold and 1.5-fold fewer TCR_1045_/Fas-4-1BB_tm_ T cells were Annexin V^+^/PI^+^ than TCR_1045_ T cells, respectively (figure 2L), suggesting both enhanced proliferation and survival of TCR_1045_/Fas-4-1BB_tm_ T cells within ID8_VEGF_ tumors.

To further interrogate the mechanism of TCR_1045_/Fas-4-1BB_tm_ T cell persistence, transcripts for the prosurvival Bcl2 protein were quantified by qPCR 8 days postactivation in vitro (figure 2M) and detected at higher levels in TCR_1045_/Fas-4-1BB_tm_ T cells relative to TCR_1045_ T cells. Intracellular staining of T cells isolated from tumors 3 days post-transfer also revealed higher Bcl2 protein expression in TCR_1045_/Fas-4-1BB_tm_ T cells (figure 2N). Together, these data suggest TCR_1045_/Fas-4-1BB_tm_ T cells achieve enhanced persistence in ID8_VEGF_ tumors by improved T cell survival and proliferation within the TME.

### TCR_1045_/Fas-4-1BB IFP^+^ T cells sustain enhanced IL2 production in solid tumors

**Figure 3:**
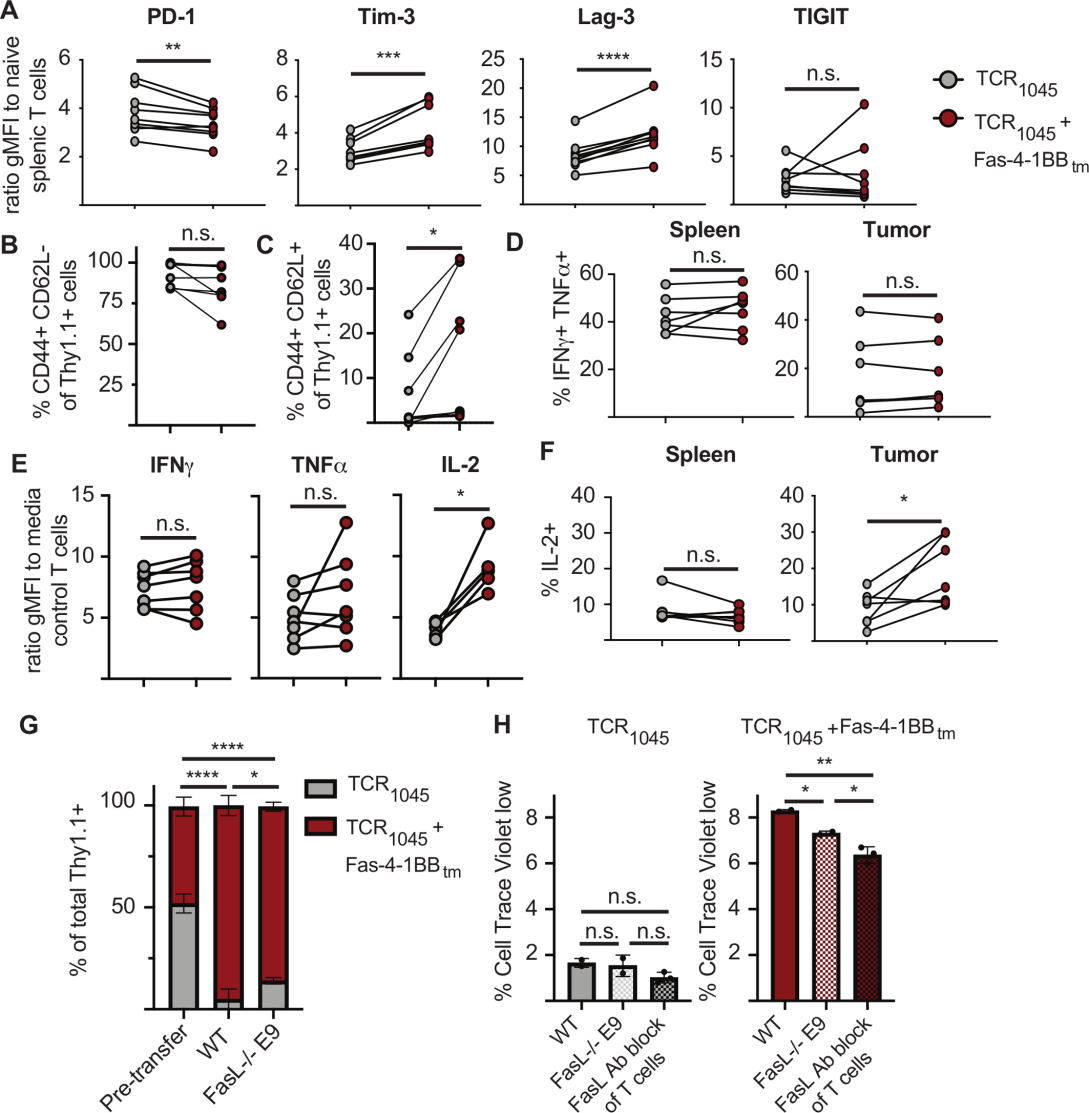
Fas-IFP-expressing T cells remain susceptible to T cell dysfunction in vivo Thy1.1^+^ or Thy1.1/1.2^+^ P14 T cells expressing TCR_1045_ or TCR_1045_/Fas-4-1BB_tm_ IFP were co-transferred at a 1:1 ratio i.p. into ID8_VEGF_ tumor-bearing mice with 5×10^7^ irradiated splenocytes pulsed with Msln_406-414_ peptide, and daily s.c. IL-2. In (E) cells were co-transferred into ID8_VEGF_ wild type or FasL^-/-^ E9 tumors. (A) at 21 days after co-transfer, T cells were isolated from tumors and evaluated by flow cytometry for expression of PD-1, Tim-3, Lag-3, and TIGIT. TCR_1045_ T cells (gray) and TCR_1045_/Fas-4-1BB_tm_ IFP^+^ T cells (red) from the same mouse are connected with a line. Data are displayed as a ratio of the geometric mean fluorescence intensity (gMFI) of Thy1.1^+^ T cells compared with endogenous CD44^-^ CD62L^+^ naïve CD8 T cells from the spleen of the same mouse. Cumulative data from two independent experiments (n=7 mice). Paired t-test. **P=0.0064; ***p=0.0005; ****p<0.0001; n.s.=not significant. (B, C) Flow cytometry staining of Thy1.1^+^ or Thy1.1/1.2^+^ donor P14 T cells for CD44 and CD62L. Data from experiments in A). Paired t-test. *P<0.05. (D, F) Ex vivo intracellular cytokine staining for IFN-γ and TNF-α or IL-2 production by P14 Thy1.1^+^ TCR_1045_ (gray) or TCR_1045_/Fas-4-1BB_tm_ IFP (red) CD8 T cells isolated from spleen or tumor, after 5 hour stimulation with Msln_406-414_ peptide. Cumulative data from two independent experiments (n=7 mice). Paired t-test. *P<0.05 (G) Proportion of donor T cells within ID8_VEGF_ wild type or FasL^-/-^ E9 tumors 7 days after co-transfer. Data pooled from three independent experiments, n=3 (WT) or n=3 (E9) mice per group. Two-way ANOVA for multiple comparisons. ****p<0.0001 (TCR_1045_ T cells pre-transfer vs WT and TCR_1045_/Fas-4-1BB_tm_ IFP T cells pre-transfer vs WT). *P<0.0474 (TCR_1045_ T cells, WT vs E9), *p<0.0345 (TCR_1045_/Fas-4-1BB_tm_ IFP T cells, WT vs E9). (H) In vitro Cell Trace Violet (CTV) proliferation assay of T cells after co-culture with wild type or FasL^-/-^ E9 ID8_VEGF_ tumor cells. Tumor cells were irradiated, pulsed with Msln_406-414_ peptide, and plated for >4 hours before adding T cells. In some conditions, T cells were pre-treated with anti-FasL blocking antibody (clone: Kay10). T cells were added at a 5:1 T cell to tumor cell ratio. Cells were co-cultured for 7 days, then stained for flow cytometry. The percent of live Thy1.1^+^ Vβ9^+^ T cells with reduced CTV staining compared with control CTV^+^ T cells cultured without tumor cells is shown. Cumulative data from two independent experiments. 1-way ANOVA for multiple comparisons. *P<0.04, **p=0.0035. ANOVA, analysis of variance.

We previously reported Fas-4-1BB_tm_ IFP-enhanced cytokine production in vitro.^13^ To evaluate if this advantage was maintained in vivo, we co-transferred P14 T cells expressing TCR_1045_ or TCR_1045_/Fas-4-1BB_tm_ into tumor-bearing mice. Twenty-one days after cell transfer, both tumor-infiltrating T cell populations expressed PD-1, Lag-3, Tim-3 and TIGIT (figure 3A, relative to naïve T cells in the spleen). Fas-4-1BB_tm_ IFP^+^ T cells expressed reduced PD-1 and elevated Tim-3 and Lag-3 inhibitory receptor proteins, relative to their TCR_1045_ counterparts. A higher proportion of intratumoral Fas-4-1BB_tm_ IFP^+^ T cells retained CD62L expression, a protein associated with a central memory phenotype, compared with TCR_1045_ T cells in the same mouse (figure 3B, C). After isolation from tumor vs spleen, a lower fraction of engineered T cells from the tumor produced the effector cytokines interferon-γ (IFNγ) and tumor necrosis factor-α (TNFα) when stimulated with MSLN_406-414_ peptide ex vivo (figure 3D), consistent with previously reported declining function in the TME.^10^ The IFP apparently does not sustain effector function in the TME as no differences were detected between TCR_1045_ and TCR_1045_/Fas-4-1BB_tm_ T cells in the fraction of cells producing these effector cytokines (figure 3D) or on a per-cell basis (figure 3E). However, the larger number of cytokine-producing IFP^+^ cells in the TME does suggest overall enhanced effector activity in IFP^+^ T cell-treated tumors. Similar to in vitro (figure 2C), a higher percentage and total number of TCR_1045_/Fas-4-1BB_tm_ vs TCR_1045_ intratumoral T cells produced IL-2 (figure 3E, F). As 4-1BB signaling induces IL-2 production by T cells,^24 25^ these data are consistent with TME-delivered FasL signals promoting sustained 4-1BB co-stimulation, thereby supporting T cell survival and proliferation. However, the data also suggest that IFP^+^ T cells are not rendered resistant to TME-mediated inhibitory signals.

### FasL signal from tumor epithelium contributes to, but is not required for, preferential accumulation of TCR_1045_/Fas-4-1BB_tm_ T cells in the TME

To determine if tumorous FasL expression is required for preferential accumulation of TCR_1045_/Fas-4-1BB_tm_ T cells, we generated tumor-bearing mice with wild type ID8_VEGF_ or E9 ID8_VEGF_^FasL-/-^ cells and co-transferred P14 T cells expressing TCR_1045_ or TCR_1045_/Fas-4-1BB_tm_. Seven days later, TCR_1045_/Fas-4-1BB_tm_ T cells preferentially accumulated in both tumors, but the enrichment of TCR_1045_/Fas-4-1BB_tm_ T cells was lower in ID8_VEGF_^FasL-/-^ tumors (figure 3G). These data suggest that FasL signaling from the tumor epithelium promotes, but is not essential for, IFP^+^ T cell persistence. To evaluate if the enhanced proliferation of TCR_1045_/Fas-4-1BB_tm_ T cells is due to FasL signaling from tumor cells and/or T cells, T cells were treated with Cell Trace Violet and co-cultured with wild type or E9 ID8_VEGF_^FasL-/-^ tumor cells for 7 days. In some conditions, T cells were pre-treated with FasL blocking Ab. ID8_VEGF_ tumor cells as stimulators induce only modest T cell proliferation. Blocking FasL from tumor cells by genetic knockout (ID8_VEGF_^FasL-/-^) or from fellow T cells by antibody blockade did not significantly impact proliferation of TCR_1045_ T cells. However, knockout of FasL from the tumor cells reduced proliferation by IFP^+^ T cells and this was further reduced when FasL was also blocked on T cells (figure 3H). Together, this data suggests that the tumor epithelium and neighboring T cells both provide FasL signals that support IFP^+^ T cell proliferation, persistence and cytokine production.

### Adoptive immunotherapy with TCR_1045_/Fas-4-1BB_tm_ T cells significantly prolongs survival of ovarian tumor-bearing mice

**Figure 4:**
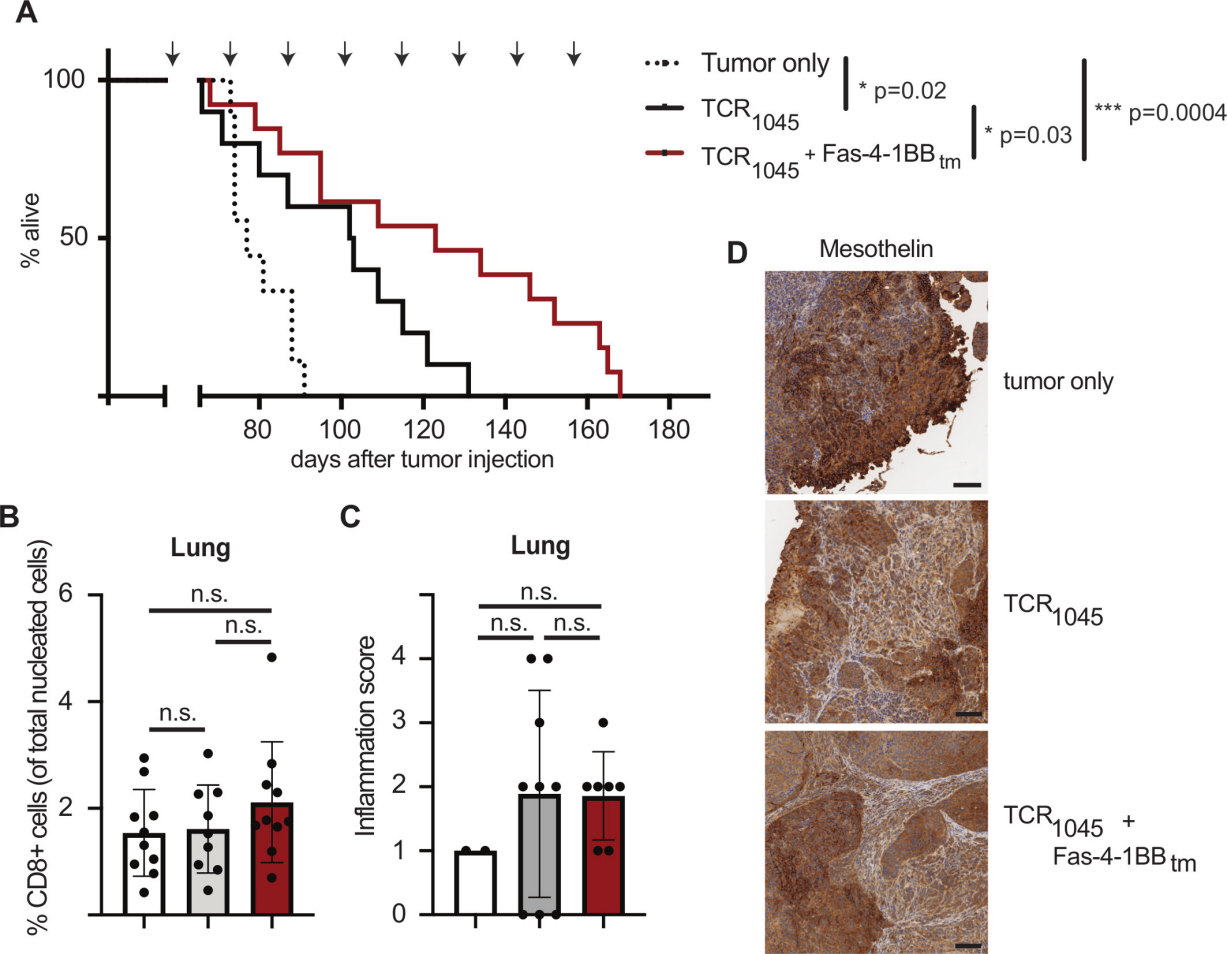
A Fas-IFP enhances antitumor efficacy in vivo (A) Overall survival of ID8_VEGF_ tumor-bearing mice treated with repeated doses of P14 Thy1.1^+^ CD8 T cells transduced with TCR_1045_ or TCR_1045_ and Fas-4-1BB_tm_ IFP. Tumor-bearing mice received a single dose of cyclophosphamide (180 mg/kg) before the first T cell infusion followed by injections of 1×10^7^ TCR_1045_ or TCR_1045_/Fas-4-1BB_tm_ T cells and peptide-pulsed irradiated splenocytes (5:1 APC:T cell ratio) i.p. every 14 days with 1×10^4^ U IL-2 s.c. for 10 days after each infusion. Treatment was initiated 45–52 days after tumor injection, when tumors were detectable by US. Arrows above survival curve indicate the timing of T cell infusions. Survival data are aggregated from three independent experiments, n=9–13 total per group. Log-rank (Mantel-Cox) test. (B) IHC quantification of CD8 +T cells in lungs of untreated (white), TCR_1045_ (gray) or TCR_1045_/Fas-4-1BB_tm_ IFP (red) treated mice at necropsy. (C) Histology scoring of hematoxylin and eosin IHC stained lungs from untreated (white), TCR_1045_ (gray) or TCR_1045_/Fas-4-1BB_tm_ IFP (red) treated mice at necropsy. All lungs contained metastatic tumor. Scoring ranged from 0 (tissue within normal limits), to 4 (neutrophilic interstitial pneumonia with or without lymphoplasmacytic and neutrophilic perivasculitis) and was performed by a trained pathologist. (B, C) Cumulative data from three independent experiments, n=9–10 per group, except the untreated mice in (C), for which only 2 mice had metastatic tumors in the lungs. One-way ANOVA with multiple comparisons. All error bars represent SD (D)Immunohistochemistry (IHC) staining for MSLN in tumors of untreated, TCR_1045_ or TCR_1045_/Fas-4-1BB_tm_ IFP treated mice at necropsy. Scale bar=100 µm. Images are representative of 9–10 mice per group from three independent experiments. ANOVA, analysis of variance; IFP, immunomodulatory fusion protein; MSLN, Mesothelin; n.s, not significant.

Evaluating whether TCR_1045_/Fas-4-1BB_tm_ T cells control progressive ovarian cancer more effectively than TCR_1045_ T cells, we used an established treatment protocol that previously revealed TCR_1045_ T cells prolong survival of ID8_VEGF_ tumor-bearing mice.^10^ Briefly, ID8_VEGF_ tumor-bearing mice were treated with a single dose of cyclophosphamide ≥6 hours prior to T cell transfer, and then received 1×10^7^ engineered T cells and 5×10^7^ irradiated splenocytes pulsed with MSLN_404-416_ peptide every 14 days (lymphodepletion occurred only before the first T cell transfer). Mice received 10^4^ U IL-2 daily for 10 days after each T cell transfer to promote T cell expansion and persistence. Mice treated with TCR_1045_/Fas-4-1BB_tm_ T cells had a significantly longer median survival (123 days) relative to control mice (77 days) or mice treated with TCR_1045_ T cells (102 days) (figure 4A).

Since MSLN is expressed on the lining of the pleural and pericardial cavities, we evaluated if IFP expression enhanced T cell accumulation/activity in the lungs. At euthanasia we evaluated CD8 T cell infiltration by IHC staining, and the proportions of intralung CD8 T cells were not statistically different between treatment groups (figure 4B), suggesting no selective accumulation of engineered T cells in lung tissues.

To assess inflammation, a pathologist evaluated H&E-stained lung and heart sections from treated and untreated mice for metastatic tumor growth and neutrophil infiltration. No treatment-related myocarditis or cardiac lesions were observed. We detected metastatic tumor growth in lungs from 2/7 untreated mice, 9/9 TCR_1045_ T cell-treated mice, and 7/12 TCR_1045_/Fas-4-1BB_tm_ T cell-treated mice, with longest surviving mice harboring the greatest number of metastatic lung nodules (excluding samples with poor fixation or staining from the analysis). The increased metastasis in treated mice may reflect the prolonged survival, with longer time for metastatic lesion development. No inflammation was observed in lungs of mice without metastatic lesions, suggesting the absence of T cell activation by normal tissues. Mice with metastatic intra-lung tumor growth were scored to determine if the presence of lung metastases increased inflammation after T cell treatment. Mild to moderate inflammation, defined as lymphoplasmacytic and neutrophilic perivasculitis, was observed in the lungs of some treated mice (figure 4C) and was scored slightly higher in treated compared with untreated mice, but this did not reach statistical significance. No mice showed signs of respiratory distress during the experimental course.

Moreover, antigen-loss immune escape was not clearly driven by TCR_1045_/Fas-4-1BB_tm_ T cells. IHC showed that MSLN expression was still present in the tumors of treated as well as untreated mice (figure 4D), consistent with the sustained upregulation of exhaustion markers we observed on tumor-infiltrating T cells (figure 3A).

### A human Fas-4-1BB IFP improves primary T cell killing of ovarian cancer

**Figure 5:**
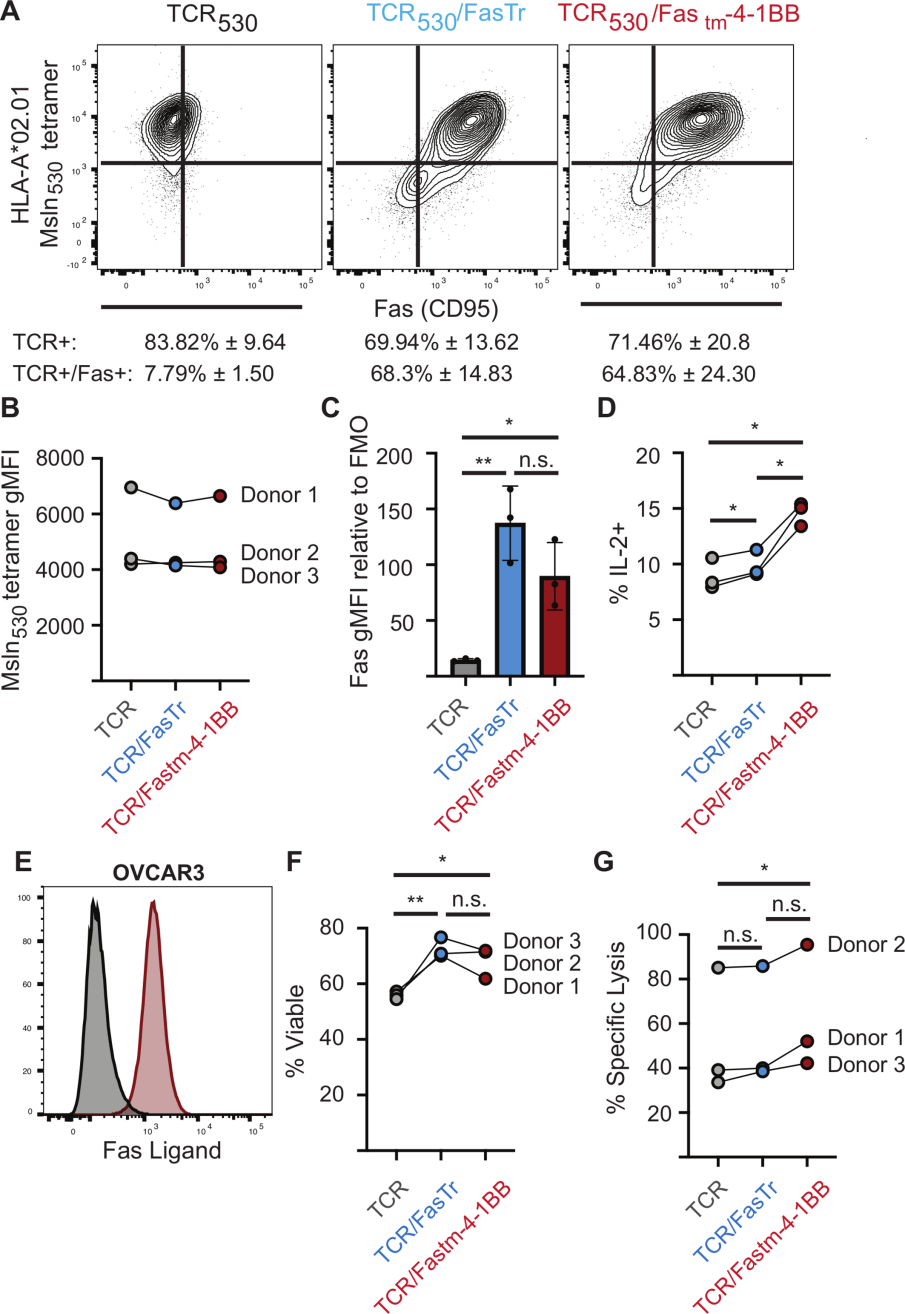
A human Fas-IFP enhances ovarian tumor cell killing Three unique donors were transduced to express an MSLN-specific TCR (TCR_530_, gray), TCR_530_ and a truncated Fas (TCR_530_/FasTr, blue) and TCR_530_ and a Fas_tm_-4-1BB IFP (TCR_530_/Fas_tm_-4-1BB, red). (A) Flow cytometry staining for HLA-A*02.01 Msln_530-538_ tetramer binding and Fas expression. Representative flow plots from one donor after two restimulations are shown. The average transduction efficiency and SD of each construct for three unique donors are listed below. (B) Geometric MFI of HLA-A*02.01 Msln_530-538_ tetramer staining for all donors. Results from each unique donor are connected with a line. (C) Geometric MFI of Fas expression relative to the fluorescence minus one (FMO) control for all donors. Analyzed with one-way ANOVA with multiple comparisons. *P=0.0283, **p=0.0028. (D) In vitro intracellular cytokine staining for IL-2 production after 5-hour stimulation with Msln_530-538_ peptide. Results from each unique donor are connected with a line. Analyzed with repeated measures one-way ANOVA for multiple comparisons. *P<0.04. (E) Flow cytometry staining for FasL on OVCAR3 tumors cells. The gray histogram represents unstained tumor cells. Representative histogram from three independent experiments. (F) Viability staining of T cells after 3 days of coculture with OVCAR3 tumor cells. Results from each unique donor are connected with a line. Analyzed with repeated measures one-way ANOVA for multiple comparisons. *p=0.0198, **p=0.0052 (G) Specific lysis of OVCAR3 cells after 155 hours of coculture with transduced T cells. Results from each unique donor are connected with a line. Analyzed with repeated measures one-way ANOVA for multiple comparisons. *P=0.025. ANOVA, analysis of variance; IFP, immunomodulatory fusion protein; ns, not significant.

To evaluate if a Fas-4-1BB IFP might improve activity against human ovarian cancer, three unique donors were transduced to express a human TCR specific for MSLN peptide 530–538 (TCR_530_) with or without a truncated Fas or a Fas-4-1BB IFP. Primary human T cells were transduced with either a bi-cistronic vector containing the alpha and beta chains of TCR_530_, a tri-cistronic vector containing TCR_530_ and a truncated Fas (FasTr), or a tri-cistronic vector containing TCR_530_ and a Fas-4-1BB fusion protein with the transmembrane domain from Fas (Fas_tm_-4-1BB). T cells were transduced and restimulated twice with irradiated feeder cells and anti-CD3 antibody and then evaluated for tetramer binding and expression of Fas (figure 5A). TCR_530_ expression varied between donors but was similar between all constructs within each donor (figure 5B). Fas expression was not statistically different between the TCR_530_/FasTr and TCR_530_/Fas_tm_-4-1BB constructs, but the truncated Fas trended toward the highest expression (figure 5C).

We previously reported that T cells expressing both TCR_530_ and a Fas-4-1BB IFP produce more IFNγ than their TCR_530_ and TCR_530_/FasTr counterparts.^13^ To determine if the 4-1BB co-stimulatory domain could promote increased IL-2 production, as observed in murine T cells (figure 2C), T cells were stimulated for 5 hours with Msln_530_ peptide. Significantly more TCR_530_/Fas_tm_-4-1BB T cells produced IL-2 than TCR_530_ and TCR_530_/FasTr T cells (figure 5D), despite slightly lower expression of the fusion protein (figure 5A, C), suggesting co-stimulatory signaling from the 4-1BB component of the IFP supports enhanced cytokine production.

To determine if the FasTr and Fas-IFP constructs reduce FasL-induced T cell death, T cells were co-cultured for 3 days with human ovarian cancer cells (OVCAR3) which express FasL (figure 5E). The co-culture period was limited to 72 hours to allow for analysis of the disruption of the death signal through dominant negative mechanisms by truncated Fas or the IFP while limiting the contribution to survival from the co-stimulatory/survival signals from the 4-1BB domain. T cells expressing FasTr or a Fas-4-1BB IFP exhibited greater viability than their TCR_530_ counterparts (figure 5F), suggesting that during this brief 3-day period of co-culture both the FasTr and IFP can similarly interfere with death from endogenous Fas signaling.

To determine if FasTr or Fas-IFP T cells can lyse human ovarian cancer cells better than TCR_530_ T cells, T cells expressing an irrelevant TCR or T cells expressing TCR_530_ with or without FasTr or a Fas-4-1BB IFP were co-cultured with OVCAR3 tumor cells 155 hours. OVCAR3 cells were allowed to adhere to E-plates overnight, T cells added, and tumor cell lysis was quantified by cellular impedance (xCELLigence, Agilent). T cells expressing TCR_530_ and the Fas_tm_-4-1BB IFP resulted in greater OVCAR3 lysis than TCR_530_ or TCR_530_/FasTr T cells (figure 5G). TCR_530_/FasTr T cells did not kill OVCAR3 tumor cells more than control TCR_530_ T cells, suggesting co-stimulation from the IFP is necessary for the improved cytolytic capability of TCR_530_/Fas-4-1BB_tm_ T cells. These results are consistent with our previous finding that the Fas-4-1BB IFP, but not the decoy Fas receptor, enhances T cell lytic ability.^13^

## Discussion

FasL/Fas signaling can mediate T cell death, including activation-induced cell death, an apoptotic mechanism that regulates T cell expansion during repeated stimulation. Tumor cells often upregulate FasL, which may protect from tumor-infiltrating lymphocytes and other inflammatory cell types. The immune-competent ID8_VEGF_ model of ovarian cancer naturally expresses FasL,^12^ providing an opportunity to interrogate strategies for overcoming this immune-evasion mechanism. We previously demonstrated that TCR_1045_ T cells could prolong survival of ID8_VEGF_ tumor-bearing mice harboring late-stage disease,^10^ but that all mice eventually succumb to disease, due at least in part to immune-suppressive mechanisms operative in the TME. CRISPR/Cas9 knock-in genetic modification of T cells with a pooled library demonstrated that a synthetic Fas-4-1BB fusion protein improved T cell fitness during in vitro expansion,^26^ and we previously demonstrated that T cells expressing a Fas-4-1BB fusion protein improved tumor cell killing in vitro and in vivo.^13^ Following transfer into ID8_VEGF_ tumor-bearing mice, we have now shown that T cells engineered to express both TCR_1045_ and a Fas-4-1BB IFP persist better in the ovarian TME than T cells modified with only TCR_1045_, and significantly further prolong host survival. We have also demonstrated that human T cells expressing a Fas-4-1BB fusion protein exhibit enhanced proliferation,^13^ cytokine production, and specific lysis of ovarian tumor cells in vitro. Thus, engineering strategies that address immune-suppressive features of solid tumors have the potential to significantly improve therapeutic benefit for patients.

FasL expression in solid tumors is heterogenous, and high expression may simultaneously support metastatic spread^17^ and immune evasion.^18^ Fas-4-1BB IFP^+^ T cells preferentially accumulated relative to IFP^-^ T cells even in E9 ID8_VEGF_^FasL-/-^ tumors, indicating that FasL expression by tumor epithelium is not required for the improved persistence of IFP^+^ T cells in situ. Since effector T cells also express FasL, the addition of a Fas-4-1BB IFP may improve therapeutic efficacy even in patients with tumors expressing low FasL levels. However, therapeutic efficacy may be greatest in tumors with high FasL expression as tumor-cell FasL expression enhanced T cell proliferation in vitro and accumulation of IFP^+^ T cells in vivo.

We previously demonstrated that IFP^+^ T cells produced more IFNγ, TNFα and IL-2 than control T cells in vitro.^13^ However, cells isolated from tumor-bearing mice and stimulated ex vivo produced similar levels of IFNγ and TNFα when isolated from tumor. Since the IFP^+^ T cells do produce more IL-2 ex vivo, have an increased frequency of cells with central memory characteristics (CD62L expression, low PD-1) and preferentially persist in tumors, these results suggest the improved tumor control observed in tumor-bearing mice may be largely due to increased persistence of tumor-specific T cells with enhanced proliferative capacity rather than enhanced cytolytic function of individual cells. These results are consistent with findings in mouse models and patients, in which T cells with a progenitor exhausted phenotype have improved persistence, proliferation and response to checkpoint blockade and correlate with improved tumor control.^14 27–29^ Thus, engineering approaches such as introducing IFPs that promote increased persistence and differentiation potential of adoptively transferred tumor-specific T cells should offer an opportunity to improve the therapeutic efficacy of cellular therapies.

Our co-transfer studies revealed that TCR_1045_/IFP^+^ T cells expressed lower levels of PD-1 but higher levels of Tim-3 and Lag-3 compared with TCR_1045_ T cells within the same TME, an unexpected finding given that inhibitory receptors are expressed in response to TCR signaling. Since expression of these molecules is regulated by distinct transcription factors, and we previously demonstrated that signaling through the IFP modulates transcription factor expression,^13^ IFP signaling may establish distinct transcription factor profiles in the two T cell populations, accounting for these differences. Ongoing single cell transcriptomic and epigenetic studies comparing TCR_1045_ and TCR_1045_/IFP^+^ T cells may reveal further insights into the regulation of these receptors.

The Fas-4-1BB IFP utilizes a decoy Fas receptor to provide co-stimulatory signals, which are often limited in solid tumors. Cell-intrinsic engineering strategies using a truncated Fas receptor can successfully disrupt death-receptor signaling and improve the efficacy of T cell therapy in a mouse melanoma model,^19^ but such dominant-negative Fas molecules still lack co-stimulatory signals that may be critical for T cell persistence in some TMEs. 4-1BB agonists, soluble 4-1BB ligand and other agents that provide systemic co-stimulation can promote anti-tumor immunity but are not cell intrinsic and can also trigger inflammation.^30^ The Fas-4-1BB IFP selectively targets co-stimulation to tumor-specific T cells, resulting in intratumoral T cells expressing more anti-apoptotic molecules and IL-2, together promoting T cell survival/proliferation and antitumor efficacy. As FasL is highly expressed in many solid tumors,^19^ and associated with poor prognosis,^11^ Fas-4-1BB IFPs may provide an opportunity to enhance engineered adoptive T cell therapy against many malignancies.

## Data Availability

Data sharing not applicable as no datasets generated and/or analysed for this study. Not applicable.
