# Supplementary figures and images for: Engineering adoptive T cell therapy to co-opt Fas ligand-mediated death signaling in ovarian cancer enhances therapeutic efficacy
Source: J Immunother Cancer. 2022 Mar 9;10(3):e003959. doi: 10.1136/jitc-2021-003959 (PMC8915280; doi:10.1136/jitc-2021-003959)

Anderson et al OC Fas Figure S1

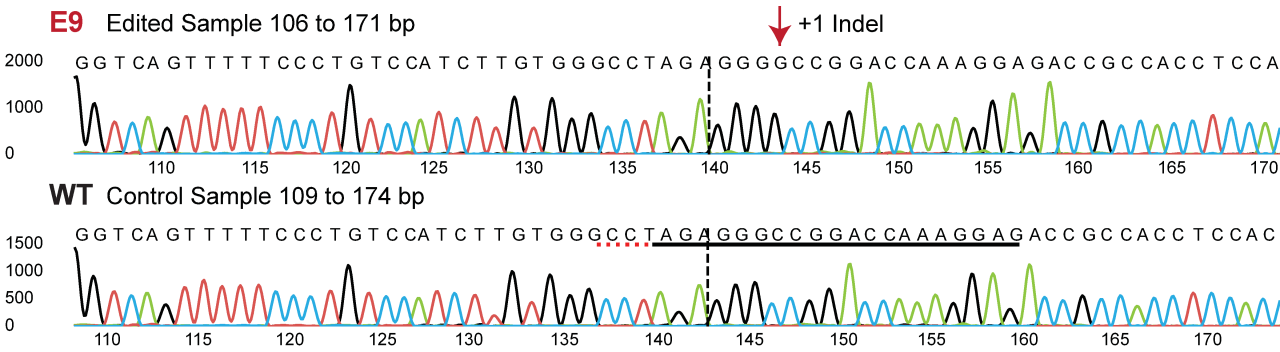

Supplement: Supplementary data [file jitc-2021-003959supp001.pdf]
